# Effect of Diabetes Mellitus on Tuberculosis Treatment Outcomes among Tuberculosis Patients in Kelantan, Malaysia

**DOI:** 10.18502/ijph.v49i8.3892

**Published:** 2020-08

**Authors:** Siti Rohana AHMAD, Nor Azwany YAACOB, Mat Zuki JAEB, Zalmizy HUSSIN, Wan Mohd Zahiruddin WAN MOHAMMAD

**Affiliations:** 1. Department of Community Medicine, Universiti Sains Malaysia, Kelantan, Malaysia; 2. Respiratory Clinic, Hospital Raja Perempuan Zainab, Kelantan, Malaysia; 3. School of Applied Psychology, Social Work, and Policy, Universiti Utara Malaysia, Kedah, Malaysia

**Keywords:** Tuberculosis, Diabetes mellitus, Tuberculosis treatment outcomes

## Abstract

**Background:**

There is growing evidence that DM may play an important role in the occurrence of unsuccessful TB treatment outcomes. This study was undertaken to examine the prevalence of DM among TB population, compare the profile of TB patients with and without DM and determine the effect of DM on unsuccessful treatment outcomes among TB patients in Kelantan state, Malaysia from 2012 to 2016.

**Methods:**

A cross sectional study was conducted in Sep 2017 using data from registered TB cases in Kelantan state, Malaysia from 2012 to 2016. The profile of TB patients with and without DM were compared in univariable analysis. Multiple logistic regression was used to determine association between DM and unsuccessful treatment outcomes.

**Results:**

A total of 1854 TB patients were diagnosed with DM. The annual proportion was ranging from 26 to 29%. TB patients with DM had an older age, live single, low educational status, poor chest x ray finding and diagnosed with smear positive sputum compared to TB patients without DM. TB patients with DM had three times higher risk to develop unsuccessful TB treatment outcomes compared to TB patients without DM (95% CI 2.47–3.58; *P* = 0.012) in multivariable analysis.

**Conclusion:**

Those with DM had the worst prognosis of TB outcomes among the significant risk factors. TB control program in Malaysia will need to expand efforts to focus on treatment of TB-DM patients to improve their cure rates in order to achieve the goals of tuberculosis elimination.

## Introduction

Tuberculosis (TB) is an infectious disease caused by *Mycobacterium tuberculosis* that is preventable and curable (1). Based on WHO surveillance and survey data, the absolute number of incident cases is declining in all six WHO regions, at an average rate of 1.5% per year from 2000 to 2013 and 0.6% between 2013 and 2015 (2). In 2016, 6.3 million new cases of TB were reported equivalent to 61% of the estimated incidence of 10.4 million. Although the incidence has been decreasing worldwide, there has been steady increased in the number of TB notification rate in Malaysia over the years (3).

Malaysia is located in South East Asia region, and categorised as an upper middle-income country, with a population size of over 32 million individuals. In 2016, the annual incidence rate of TB is 92 per 100,000 populations (2). The southeast region, of which Malaysia is part, accounted for nearly half of the global burden in terms of new TB cases appearing each year. Three of the 22 countries with the world’s highest TB burden (Indonesia, the Philippines and Thailand) are neighbouring countries (4).

Diabetes Mellitus (DM) is known to be one of the risk factor for TB (5). The developing countries experienced an increase in the total number of DM patients over the years. In Malaysia, the prevalence has been rising from 11.6% in 2006 to 17.5% in 2015 (6). Based on the results of the National Health and Morbidity Survey or NHMS I (1986), NHMS II (1996), NHMS III (2006), the projection of DM prevalence will be 15.3% by year 2020 but it has reached the projected level nine yr earlier (6,7). The revised projection will be 21.6% with an estimated 4.5 million Malaysians age 18 yr and above are expected to have DM by year 2020 (6). The convergence of this two epidemic enhances the chances of an escalating number of TB patients with DM in the future (5,7).

A strong association between DM and TB disease was reported in previous studies. DM weaken person’s immune system and increase the risk to get active TB. During TB treatment, people with DM have 2 times higher risk of remaining culture positive, 4 times higher risk of relapse after completed treatment and 5 times higher risk of death, as compared to those without DM (8–10). The poor TB treatment outcomes became a major threat to global TB control programme (11).

To our knowledge, published studies looking at association between TB and DM in Malaysia are still lacking. Recent study in Malaysia had reported a positive association between DM and poor TB treatment outcomes (12). However, the trend might not be same in general population since it was a hospital-based study. Further clarification and quantification of association between DM and these poor TB treatment outcomes in our community is highly needed for better understanding and planning. Assessing the status of local setting by understanding socio demographic, clinical characteristic and risk factor will provide local data that are considerably useful in evaluating the effectiveness of management as well as preventive control strategies and intervention for TB patients with DM.

We therefore conducted a study to examine the prevalence of DM among TB population, compare socio demographic and clinical characteristic among TB patients with and without DM and determine the effect of DM on unsuccessful TB treatment outcomes in Kelantan state, a northeast coast of peninsular Malaysia.

## Materials and Methods

Kelantan, is located in the northeast coast of Peninsular Malaysia, facing the South China Sea in the northeast and bounded by Thailand in the north, Terengganu in the east; Pahang in the south; and Perak in the west. This state has 10 administrative divisions with a population of 2 million and has implemented Government of Malaysia Revised National Tuberculosis Control programme since 2011. All cases of TB diagnosed according to Malaysian Tuberculosis guideline were registered into Malaysian National Tuberculosis Information System (MyTB). MyTB is a web-based TB registration database that provide data information of all registered TB cases in Malaysia that considered a fairly consistent and complete data source.

This was a cross sectional study conducted in Sep 2017 using data from registered TB cases identified in MyTB from 1st Jan 2012 to 31^st^ of Dec 2016. The information includes patient’s sociodemographic (age, race, gender, marital status, nationality, residence, education status, employment status, smoking status), clinical characteristics (comorbid disease, presence of BCG scar, mantoux test, laboratory test, HIV status, chest x-ray finding, history taking, TB regime, patient’s information for DOT) and TB treatment outcomes. DM status in MyTB was reported as dichotomous variable (no/yes) and defined as presence of confirmation by measurement of venous plasma glucose. In current Malaysian setting, the DM screening before initiation of anti-TB drugs is mandatory. Confirm DM cases is when fasting venous plasma glucose is 7.0mmol/L and more and random blood sugar is 11.0 mmol/L and more. The diagnostic values is followed the ministry of health guideline (13).

Based on the Malaysian TB treatment guideline, TB treatment outcomes after six months of TB regimens were classified as cured, completed, failed, died, treatment interrupted, loss to follow up, change diagnosis and ongoing treatment. The treatment outcomes were further classified as successful (cured and completed treatment), or unsuccessful (died, failed treatment, loss to follow up, changed diagnosis, ongoing treatment and treatment interrupted).

Data were entered, cleaned and analysed using Statistical Package For Social Science (SPSS) version 22 Software. The sociodemographic, clinical characteristics and TB treatment otcomes of TB patients were described and compared between TB patients with and without DM using Chi Square test and Independent t-test. Sociodemographic variables were age, gender, races, marital status, nationality, educational level, employment status, residence and smoking status. Clinical characteristics variables were type of TB, case categories, sputum smear, HIV status and chest x-ray finding. TB treatment outcomes were categorised into cured, completed, failed, died, treatment interrupted, loss to follow up, change diagnosis and ongoing treatment. Final TB treatment outcomes were classified into successful (cured and completed treatment) and unsuccessful (died, failed treatment, change diagnosis, loss to follow up, ongoing treatment and treatment interrupted). All categorical variables were described in frequency and percentage. Numerical data were described in mean and standard deviation (SD) or median and interquartile range (IQR) depending on normality of distribution. Multiple Logistic Regression (MLogR) was used to to determine the effect of DM on unsuccessful TB treatment outcome. The outcome was a binary variable coded “0” for successful and “1” for unsuccessful. Variables with *P*-value of less than 0.25 from univariable analysis and clinically important were selected for MlogR. Preliminary main effect model was obtained after comparing model using forward LR and backward LR methods. Multicol-linearity was checked by using correlation matrix in which if the correlation between variables were weak it indicated no multicollinearity. Variance Inflation Factor (VIF) was also used to check multicollinearity, valued in which showed less than 10 was acceptable and indicated no multicollinearity problem. All possible two - way or first order interaction were checked. Then, preliminary final model was obtained. Fitness of model was tested by Hosmer and Lemeshow goodness of fit test. The classification table and receiver operator characteristic (ROC) curve were also used to determine the fitness of the model. The Hosmer and Leme- show goodness of fit test with *P*-value more than 0.05 which was not significant indicated model was fit. Classification table showed more than 70% was considered a good model. The area under the curve above 0.7 with *P*-value less than 0.05 also indicated that model was fit. The final model was determined by enter method. It was presented with adjusted odds ratio and 95% confidence interval, Wald statistics and *P*-value. The level of significance was set at *P*-value of less than 0.05.

### Ethical approval

Confidentiality was well kept during this study using anonymous technique, in which only researcher that was able to assess the name of patients. Ethical clearance approval was obtained from Research Ethics Committee (Human), Universiti Sains Malaysia on 12 Feb 2017, National Institute of Health and Medical Research Ethics Committee (MREC), Malaysian Ministry of Health on 28 Dec 2016.

## Results

Overall 6689 registered TB cases from 2012 to 2016 were retrieved from Malaysian National Tuberculosis Information system (MyTB). From these, 31 were excluded due to transferred in case from outside Kelantan. As a result, 6658 patients were included in final analysis. Our study found the annual proportion of TB patients with DM from 2012 to 2016 are increasing in Kelantan (Table 1).

**Table 1:** TB patients with and without DM registered in Kelantan state, Malaysia (2012–2016)

***Characteristics***	***2012 n(%)***	***2013 n(%)***	***2014 n(%)***	***2015 n(%)***	***2016 n(%)***
All TB patients	1410	1403	1361	1233	1251
TB with DM	377(26.74)	382(27.23)	382(28.06)	347(28.14)	366(29.26)
TB without DM	1033(73.26)	1021(72.77)	979(71.94)	886(71.86)	885(70.74)

For sociodemographic characteristics, TB patients with DM were more likely to be older, single, employed and have low educational level (primary school and below) compared to TB patients without DM (Table 2). In clinical characteristics, a significant difference seen between TB patients with and without DM in term of sputum smear, chest x-ray finding, case categories and HIV status factor (Table 3). The overall TB treatment success rate for TB patients with DM was 81%. The number of TB patients who failed treatment was higher in DM patient compared to patients without DM (Table 4). Death was the major reason of unsuccessful TB treatment outcome.

**Table 2:** Socio demographic characteristics of TB patients with and without DM, in Kelantan state, Malaysia, 2012–2016

***Characteristics***	***Total n(%)*** ***n=6658***	***TB with DM n(%)*** ***n=1854***	***TB without DM n(%)*** ***n=4804***	***P-value^a^***
Age	48.12±10.24	56.35±11.38	42.57±18.53	<0.001^b^
Gender				
Female	2237(33.60)	645(34.78)	1592(33.14)	0.21
Male	4421(66.40)	1209(65.22)	3212(66.86)	
Race				
Malay	6179(92.81)	1761(94.98)	4418(91.97)	0.872^c^
Chinese	118(1.77)	14(0.76)	104(2.17)	
Indian	11(0.17)	3(0.16)	8(0.17)	
Peninsular indigenous	109(1.64)	4(0.21)	105(2.19)	
Sabah indigenous	6(0.09)	0(0)	6(0.12)	
Others	58(3.52)	10(3.89)	48(3.38)	
Nationality				
Malaysian	6540(98.23)	1840(99.25)	4700(97.84)	<0.001
Non Malaysian	118(1.77)	14(0.75)	104(2.16)	
Marital status				
Single	2363(35.49)	987(53.23)	1376(28.64)	0.68
Married	4295(64.51)	867(46.77)	3428(71.36)	
Education status				
No formal education	898(13.49)	278(14.99)	620(12.90)	0.57
Primary school	1291(19.39)	486(26.21)	805(16.76)	
Secondary school	3620(54.37)	908(48.96)	2712(56.45)	
College/University	849(12.75)	182(9.84)	667(13.89)	
Employment status				
Unemployed	223(3.35)	58(3.13)	165(3.43)	0.98
Self employed	1035(15.55)	330(17.80)	705(14.66)	
Employed	4797(72.04)	1443(77.83)	3354(69.81)	
Prisoners	92(1.38)	20(1.09)	72(1.50)	
Student	511(7.68)	3(0.15)	508(10.6)	
Residence				
Urban	1377(20.68)	394(21.25)	983(20.46)	0.76
Rural	5281(79.32)	1460(78.75)	3821(79.54)	
Smoking status				
No	4019(60.36)	1213(65.43)	2806(58.41)	0.002
Yes	2639(39.64)	641(34.57)	1998(41.59)	

a Chi Square Test //

b Independent t-test //

c Fisher exact test

**Table 3:** Clinical characteristics of TB patients with and without DM Mellitus, Kelantan state, Malaysia, 2012–2016

***Characteristics***	***Total n(%)*** ***n=6658***	***TB with DM n(%)*** ***n=1854***	***TB without DM n(%)*** ***n=4804***	***P-value^a^***
HIV status				
Negative	6434(96.64)	1797(97.00)	4637(96.50)	0.021
Positive	73(1.09)	8(0.40)	65(1.40)	
Not done	151(2.27)	49(2.60)	102(2.10)	
Chest x-ray				
No lesion	677(10.20)	102(5.50)	575(12.00)	0.032
Far advance	163(2.40)	33(1.80)	130(2.70)	
Moderately advance	1980(29.70)	640(34.50)	1340(27.90)	
Minimal	3752(56.40)	1063(57.30)	2689(56.00)	
Not done	86(1.3)	16(0.90)	70(1.50)	
Type of TB				
Extra pulmonary	966(14.50)	129(7.00)	837(17.40)	0.671
Pulmonary	5460(82.00)	1697(91.50)	3763(78.30)	
Both	232(3.50)	28(1.50)	204(4.20)	
Case categories				
New case	6076(91.30)	1704(91.90)	4372(91.00)	0.006
Retreatment	582(8.70)	150(8.10)	432(9.00)	
Sputum smear status				
Smear negative AFB	2622(39.40)	521(28.10)	2101(43.70)	0.042
Smear positive AFB	3838(57.60)	1304(70.30)	2534(52.70)	
Not done	198(3.00)	29(1.60)	169(3.50)	

**Table 4:** Characteristics of treatment outcomes among TB patients with and without DM, Kelantan state, Malaysia, 2012–2016

***Characteristics***	***Total n(%)*** ***n=6658***	***TB with DM n(%)*** ***n=1854***	***TB without DM n(%)*** ***n=4804***	***P-value^a^***
Treatment outcomes				
Successful	5149(77.33)	1511(81.49)	3638(75.71)	
Cured	3162(47.49)	1129(60.89)	2033(42.31)	0.004
Completed	1987(29.84)	382(20.60)	1605(33.40)	<0.001
Unsuccessful	1509(22.67)	343(18.51)	1166(24.29)	
Failed treatment	10(0.15)	7(0.38)	3(0.06)	0.531
Died	773(11.61)	196(10.57)	577(12.01)	0.001
Treatment interrupted	321(4.82)	60(3.23)	261(5.43)	0.822
Loss to follow up	40(0.60)	3(0.16)	37(0.77)	0.018
Ongoing treatment	100(1.50)	22(1.20)	78(1.65)	0.156
Changed diagnosis	265(3.99)	55(2.97)	210(4.37)	0.134

a Chi-Square test

In multivariable analysis, a TB patient with DM has 3.5 higher odds to develop unsuccessful treatment outcome compared to TB patients without DM (Table 5) (AOR 3.48, 95% CI 2.47– 3.58; *P* = 0.012). The other significant factors that associated with unsuccessful TB treatment outcomes were HIV positive patients (AOR 14.35, 95% CI: 1.61–127.62; *P* = 0.017), retreatment cases (AOR 2.53, 95% CI: 1.43–4.49; *P* = 0.002), smoker (AOR 1.89, 95% CI: 1.27–2.82; *P*= 0.002), and advance chest x-ray findings (AOR 1.64, 95% CI: 1.09–2.47; *P* = 0.019) when other variables were controlled.

**Table 5:** Effect of DM on unsuccessful treatment outcome (n=6658)

***Factors***	Crude OR ^*^(95% CI)	Adjusted OR ^**^(95% CI)	***Wald statistic (df)***	P-value^**^
Case categories				
New case	1.00	1.00		
Retreatment	2.65(1.51, 4.64)	2.53(1.43, 4.49)	10.06(1)	0.002
Diabetes Mellitus				
No	1.00	1.00		
Yes	3.51(2.55,4.02)	3.48(2.47,3.58)	11.21(1)	0.012
Smoking				
Non smoker	1.00	1.00		
Smoker	1.94(1.30, 2.85)	1.89(1.27, 2.82)	9.80(1)	0.002
Chest X ray				
Minimal	1.00	1.00		
Advance	1.64(1.09, 2.47)	1.64(1.09, 2.47)	5.52(1)	0.019
HIV Status				
Negative	1.00	1.00		
Positive	13.45(1.56,116.13)	14.35(1.61, 127.62)	5.71(1)	0.017

* Simple logistic regression

** Multiple logistic regression Hosmer and Lemeshow test, (*P*-value = 0.527)

Classification table (overall correctly classified percentage = 73.4 %) Multicollinearity and interaction term were checked and not found

Area under ROC curve was (65.4%) were applied to check the model fitness

## Discussion

Our data seem to confirm an increased prevalence of DM in TB populations. About 26–29% of TB patients were identified with DM annually. Although TB incidence is decreasing, the diabetics among TB cases increased significantly and progressively for 5 years period between 2012 and 2016. The proportion was higher compared to the study’s findings from other parts of the world (5, 14–16).

In general, the patients’ sociodemographic and clinical characteristics were almost similar to the TB patients with DM in the existing studies of Malaysia and other countries (5,10). Upon comparison of this work with those studies, it was observed that most patients were older in age. Our result supported the findings reported by the studies in other countries whereby the mean age of TB patients with DM was 55 yr, while the mean age of TB patients without DM was 40 yr (13, 17–20). This finding may be attributable to the fact that the older adults are at a higher risk of developing type 2 DM due to the combined effects of increased insulin resistance, pancreatic islet dysfunction, and lifestyle changes. Both ageing and DM compromise the immune system of the hosts, increasing their susceptibility to infections such as TB (21).

It was observed that majority of TB patients with DM were single. The results also consistent with the studies carried out in India, Taiwan, Mexico, Indonesia, and China (14, 22–25). One of the reasons is single patient have less social support and had poor blood glucose control that made them susceptible to get the infection. Single person also is commonly involved in unhealthy risk behaviour like alcohol abuse and smoking that can faster the disease progression and severity (12).

In relation to the clinical characteristics, this study observed a higher frequency of pulmonary cavities in the TB patients with DM which corresponded to other studies (5, 7–12). This finding was also supported by several radiological studies which indicated that the ‘non-diabetic TB’ affects upper lobes with mild pulmonary infiltrates, cavitary lesions or hilar lymphadenopathy (8–10). Meanwhile, the ‘diabetic TB’ presents with more extensive and confluent lesions, involving multiple lobes and more of the parietal pleura and forming cavities more frequently (11–12). Nevertheless, Alishjahbana et al., (14) reported opposite findings whereby the TB patients with DM were more likely to develop mild clinical symptoms during their initial presentation to the healthcare facilities.

In this present study, the overall success rate in TB patients with DM was higher compared to TB patients without DM. This might explain the possibility of extra care and effectiveness of the dual management of patients with co morbidity in the current health system. However, this number is still not achieving the 85% target rate set by WHO (2). Meanwhile, the presence of DM seems to worsen prognosis of TB. DM demonstrate a strong association with unsuccessful TB treatment outcome among the significant risk factors. The possible explanation is due to impaired cell mediated immunity in DM patients and poor glycaemic control (16). This study finding was in agreement with other studies that reported DM as independent factor for unsuccessful TB treatment outcome (8, 14, 16, 18, 26–28).

To our knowledge, this study is the first published study from Kelantan in Malaysia assessing the association between DM and unsuccessful TB treatment outcomes. The strengths of this study are its big sample size and the utilisation of data based on an electronic information system that fairly considered a complete and consistent data based. Under National Tuberculosis Program, bidirectional screening of TB and DM were implemented in Mar 2016. The TB patients were mandatorily screened for DM before initiation of TB treatment. Diagnosis of DM was confirmed by repeated measurement of fasting blood sugar (FBS) or oral glucose tolerance test (OGTT) and it followed the current national guideline. This results in no missing in detection of newly and known diabetics in TB population which makes the data is more reliable and representative the TB and DM situation in the state and in the country.

### Limitation

First, this study did not measure the level of blood glucose control since the DM information in our data based is only reported as dichotomous variable (no/yes) without mentioned about the nature of DM development, control and medication. Therefore, the effect of DM control on TB treatment outcomes could not be measure. Second, this study was a cross sectional study with no demonstration of temporal relationship. Therefore, we cannot ascertain or speculate whether DM worsen TB or vice versa and both can be possible. A further study will be conducted to examine the temporal relationship between DM control and its impact on TB treatment outcomes.

## Conclusion

Those with DM had the worst prognosis of TB outcomes among the significant risk factors. The findings of this study underline the need of frequent monitoring and improve the care of patients with concomitant TB and DM.

## Ethical considerations

Ethical issues (Including plagiarism, informed consent, misconduct, data fabrication and/or falsification, double publication and/or submission, redundancy, etc.) have been completely observed by the authors.
